# Phenotype Assessment and Putative Mechanisms of Ammonium Toxicity to Plants

**DOI:** 10.3390/ijms26062606

**Published:** 2025-03-13

**Authors:** Lin-Bei Xie, Li-Na Sun, Zhong-Wei Zhang, Yang-Er Chen, Ming Yuan, Shu Yuan

**Affiliations:** 1 College of Resources, Sichuan Agricultural University, Chengdu 611130, China; kkskado@163.com (L.-B.X.); aasylvi@163.com (L.-N.S.); zzwzhang@126.com (Z.-W.Z.); 2 College of Life Science, Sichuan Agricultural University, Ya’an 625014, China; anty9826@163.com (Y.-E.C.); yuanming@sicau.edu.cn (M.Y.)

**Keywords:** ammonium toxicity, SnRK1, C-N metabolism, rhizosphere acidification, ion imbalance, phytohormone homeostasis

## Abstract

Ammonium (NH_4_^+^) and nitrate (NO_3_^−^) are the primary inorganic nitrogen (N) sources that exert influence on plant growth and development. Nevertheless, when NH_4_^+^ constitutes the sole or dominant N source, it can inhibit plant growth, a process also known as ammonium toxicity. Over multiple decades, researchers have shown increasing interest in the primary causes, mechanisms, and detoxification strategies of ammonium toxicity. Despite this progress, the current investigations into the mechanisms of ammonium toxicity remain equivocal. This review initially presents a comprehensive assessment of phenotypes induced by ammonium toxicity. Additionally, this review also recapitulates the existing mechanisms of ammonium toxicity, such as ion imbalance, disruption of the phytohormones homeostasis, ROS (reactive oxygen species) burst, energy expenditure, and rhizosphere acidification. We conclude that alterations in carbon–nitrogen (C-N) metabolism induced by high NH_4_^+^ may be one of the main reasons for ammonium toxicity and that SnRK1 (Sucrose non-fermenting 1-related kinase) might be involved in this process. The insights proffered in this review will facilitate the exploration of NH_4_^+^ tolerance mechanisms and the development of NH_4_^+^-tolerant crops in agricultural industries.

## 1. Introduction

Nitrogen (N) is a crucial macronutrient for plants and plays a significant role in influencing crop yields and characteristics [1]. It is a fundamental constituent of proteins, nucleic acids, chlorophyll, and enzymes [1,2]. The productivity of crops is greatly dependent on the application of nitrogen fertilizers [1,2]. The manufacturing and utilization of nitrogen fertilizers require substantial energy consumption, and an overabundance of nitrogen generates adverse effects on the environment [1,2]. Therefore, enhancing the N use efficiency (NUE) of plants is imperative for the advancement of sustainable agricultural industry [2].

In agriculture, both nitrate (NO_3_^−^)- and ammonium (NH_4_^+^)-based fertilizers are widely employed [2,3]. The absorption of NH_4_^+^ or NO_3_^−^ by higher plants mainly occurs through AMTs (ammonium transporters) and NRTs (nitrate transporters), respectively. The uptake of NH_4_^+^ at high concentrations is achieved via the non electrogenic influx of NH_3_ across the plasma membrane (PM) by diffusion, with H^+^ remaining in the apoplast. NH_3_ is reprotonated to form NH_4_^+^ after entering the root cells [4,5]. The NO_3_^−^ transmembrane transport is an active process promoted by the H^+^ potential derived from the action of PM H^+^-ATPase and requires energy, while the transport of NH_4_^+^ is a passive process and requires less energy [2,6,7]. Thus, when both NO_3_^−^ and NH_4_^+^ are present at the same time, plants are more likely to absorb NH_4_^+^ because of the lower energy expenditure [8,9]. However, as the preferred N source for plants, an excessive amount of NH_4_^+^ as the main or sole N source will lead to retardation in plant growth [10]. The large-scale application of ammonium fertilizer over recent decades has led to serious acidification of large areas of farmland [11] and inhibited the soil nitrification rate [12]. It affects plant growth and nitrogen uptake [13,14], and causes the local disappearance of NH_4_^+^-sensitive species of trees, grasses, aquatic plants, and even fishes [15]. For example, a recent investigation demonstrated that NH_4_^+^ exposure inhibits the growth of lettuce, a widely consumed vegetable [16]. Additionally, with the concentration of CO_2_ in the atmosphere likely to double by the end of this century due to its current rise from 322 ppm in 1969 to >400 ppm today [17], elevated CO_2_may lead to inhibition of NO_3_^−^ uptake rather than NH_4_^+^ uptake [18].

Thus, it is of paramount significance to investigate the mechanisms of ammonium toxicity for the sustainable development of agriculture. Nevertheless, existing studies have only demonstrated that the causes of ammonium toxicity mainly encompass rhizosphere acidification, ion imbalance, reactive oxygen species(ROS) accumulation, excessive energy consumption, etc. [1,10,15]. However, none of these are the direct reasons for ammonium toxicity. A large number of proteins and genes are implicated therein, but how these molecules respond to high NH_4_^+^ stresses remains ambiguous. On the other hand, the main results of ammonium toxicity also remain elusive, as most previous studies have paid attention to root morphological change, but not growth inhibition and developmental arrest of the whole plant seedling. In this review, we mainly focus on the mechanisms and mitigation measures to ammonium toxicity and present a comprehensive assessment system for the symptoms of ammonium toxicity.

## 2. Comprehensive Evaluation System of Symptoms in Ammonium Toxicity

Researchers have indicated that the common symptoms of ammonium toxicity in most plant species include hindered plant growth, alterations in root structure, and leaf chlorosis [10,19,20,21,22,23]. Nevertheless, most studies on this subject remain limited in quantification to root morphological changes. Therefore, the development of a systematic approach for assessing ammonium toxicity is necessary.

High ammonium can induce significant alterations in root structure through intricate mechanisms. High NH_4_^+^ hinders phloem function and impedes primary root elongation due to inadequate sucrose distribution to the root growth zone [24]. However, no notable changes in lateral root development were detected in the above report, which may be attributed to the relatively low ammonium concentration (5 mM) used by the authors [24]. Root elongation is closely associated with the auxin (Indole Acetic Acid, IAA) level, as Di and co-authors identified that elevated NH_4_^+^ levels inhibited root elongation via an auxin transporter PIN5-mediated pathway [25,26]. Previous studies also indicated that ammonium toxicity can lead to modifications in lateral roots, reduced root/shoot ratios, and diminished fresh root weight [21,22,27,28]. When evaluating the effects of ammonium toxicity on plant roots, multiple indexes, such as primary root elongation, lateral root growth and development, root/shoot ratio, and fresh root weight, should all be taken into consideration.

Moreover, ammonium toxicity also impacts stem growth, as evidenced by Hachiya et al. [21], who observed a significant reduction in fresh stem weights of wild type plants under high ammonium stress. While *ammonium-insensitive 2* (*ami2*)mutants exhibited higher stem fresh weights compared to the wild type. Similarly, Li et al. [29] demonstrated that ammonium toxicity inhibited stem growth.

In addition to the roots and stem, leaf development is also significantly hindered under ammonium stresses [10]. An early study showed that in *Arabidopsis thaliana*, ammonium stress was observed to trigger a chloroplast retrograde signal leading to leaf chlorosis, whereas activating abscisic acid (ABA) signaling could prevent leaves from chloroplast damage [30]. Furthermore, defects in Dolichol Phosphate Mannose Synthase 1 (DPMS1)also resulted in both leaf chlorosis and the inhibition of root elongation in the presence of excess NH_4_^+^ [31]. Besides Arabidopsis, ammonium stress can also cause chlorosis of barley leaves [32]. Similarly, recent studies also showed that leaf growth and development were inhibited under ammonium toxicity [21,29]. 

Furthermore, high ammonium stress has been shown to significantly reduce plant biomass and yield [21,22,33,34,35,36]. And high NH_4_^+^ induced late flowering [37]. And in some conditions, high ammonium can even lead to death of the whole plant seedling [4,10,38] (Table 1).

The multifaceted effects of ammonium toxicity on plant growth and development necessitate a thorough evaluation encompassing a wide array of indicators, rather than a narrow focus on roots. Here we suggest that both root phenotypes and shoot phenotypes should be considered when determining the severity of ammonium toxicity. Otherwise, we may identify some mutants that are only related to root development, but not to ammonium toxicity directly.

## 3. Putative Mechanisms of Ammonium Toxicity

### 3.1. Alleviation of Ammonium Toxicity in NO_3_^−^-Dependent Pathway

The ratio of NO_3_^−^ and NH_4_^+^ plays a significant role in mitigating ammonium toxicity in various plant species, including maize, wheat, tomato, cucumber, and Arabidopsis [39,40,41,42]. This phenomenon, known as NO_3_^−^-dependent alleviation of NH_4_^+^ toxicity, demonstrates that a minimal presence of NO_3_^−^, as low as 0.1 mM, can effectively counteract the toxic effects of 5–10 mM NH_4_^+^ in plants [39,40,41,42]. Sun et al. [22] and Zheng et al. [34] found that increasing the concentration of NO_3_^−^negated the ammonium toxicity phenotypes of the *slah3* (*Slow anion channel-associated 1 homologue 3*) mutant. These studies further elucidated that the exogenous application of NO_3_^−^ or the efflux of NO_3_^−^ by SLAH3 significantly alleviated the ammonium toxicity. The nitrate transporterNRT1.1 is also involved in this process. Under high ammonium stress, NRT1.1 facilitates the co-inward transport of NO_3_^−^/H^+^, leading to a reduction in extracellular H^+^ concentration and inhibition of rhizosphere acidification, thereby alleviating toxicity [36]. However, due to the low extracellular NO_3_^−^ concentration, sustained inhibition of rhizosphere acidification is not achieved. Consequently, plants utilize SLAH3 to enhance the efflux of intracellular NO_3_^−^ to maintain a high activity of NRT1.1-mediated co-inward transport of NO_3_^−^/H^+^. Through synergistic activation of the NRT1.1-SLAH3 complex, an efficient transmembrane cycling of NO_3_^−^ is induced, effectively inhibiting rhizosphere acidification and mitigating ammonium toxicity [36].

NO_3_^−^ can also mitigate ammonium toxicity by regulating rhizosphere and intracellular pH and enhancing ammonium assimilation in *Brassica napus* [43], suggesting that NO_3_^−^ alleviates ammonium toxicity primarily through the inhibition of rhizosphere acidification [42,44,45].

In addition, NO_3_^−^ may function as a signaling molecule to modulate the signal pathways of phytohormones. For instance, a low level (0.1 mM) of NO_3_^−^ was sufficient to enhance IAA and cytokinin levels but decrease ABA levels under high-NH_4_^+^ (5 mM) conditions [40] (Table 2).

However, the key genes identified in the above studies maybe merely related to nitrate absorption/transport, rather than directly related to ammonium toxicity. The mechanisms of toxicity caused by a high ratio of ammonium to nitrate and by a solely ammonium treatment may be significantly different. We must pay attention to this discrimination in future research. In the following discussion, we only focus on toxic mechanisms of the sole ammonium treatment.

### 3.2. High NH_4_^+^-Induced ROS Accumulation

Ammonium toxicity, as an abiotic stress, can induce perturbations in ROS homeostasis in vivo [4]. Li et al. [30] observed a significant increase in hydrogen peroxide (H_2_O_2_) accumulation within chloroplasts under ammonium stress. And a recent study has demonstrated that high NH_4_^+^ leads to dysregulation of C-N metabolism, impairment of the photosynthetic electron transport chain, and excessive ROS accumulation in oilseed rape leaves [35]. Liu et al. [24] found that a solely ammonium treatment triggered the generation of ROS, resulting in callose deposition and disruption of phloem function. The application of exogenous ROS scavengers, such as dimethyl thiourea (DMTU) and 4-hydroxy-TEMPO (TEMPO), significantly attenuated the deposition of ROS [24]. However, using some other ROS scavengers, like ascorbic acid (ASA) and glutathione (GSH), may lead to stunted plant growth, possibly due to their potential to enhance hydroxyl radical production under Fe-rich conditions through the Fenton reactions [46]. A recent study demonstrated that vitamin B6 was involved in the regulation of ammonium toxicity [47]. High NH_4_^+^ triggered the accumulation of ROS in roots, particularly an enrichment of H_2_O_2_ in the extended and mature areas of root tips. The synthetic deficient mutant of vitamin B6exhibited heightened sensitivity to ammonium exposure. Moreover, supplementation with exogenous vitamin B6 or enhancement of endogenous synthetic vitamin B6 significantly bolstered root tolerance to ammonium [47]. VB6 is an antioxidant compound that can alleviate the toxic effects induced by high ammonium [47].

In addition to exogenous ROS scavengers, peroxidase also contributes to the elimination of excessive ROS induced by high ammonium [48]. Superoxide dismutase (SOD), ascorbate peroxidase (APX), and catalase (CAT) also play pivotal roles in the antioxidant system [48].

However, the mechanism by which high ammonium activates an ROS signal remains inadequately elucidated. Significant enhancement of respiratory activity has been observed upon exposure to high NH_4_^+^ concentrations. Ammonium supplies the necessary energy and carbon skeletons essential for NH_4_^+^ transport and assimilation in the roots. Nevertheless, the modified respiratory activity in plants supplied with NH_4_^+^ may induce mitochondrial ROS production, thereby inducing another kind of oxidative stress [49] (Table 2).

Nitrate reductase, through the mitochondrial amidoxime reducing component (mARC), has been shown to produce the second messenger nitric oxide (NO) from nitrate, an important general regulator [50,51]. High levels of NH_4_^+^ induced NO accumulation and stimulated the accumulation of GSNOR (S-nitrosoglutathione reductase) in roots. GSNOR over-expression improved root tolerance to NH_4_^+^, while loss of GSNOR further induced NO accumulation, and enhanced root growth sensitivity to NH_4_^+^ [23].

### 3.3. High Ammonium-Induced Ion Imbalance

NH_4_^+^ can compete with and inhibit the uptake of essential cations, which are important for signal transduction and activities of a number of key enzymes [36]. A recent study revealed a new mechanism by which ammonium toxicity may induce iron (Fe)accumulation and itssubsequent adverse effects [25]. Under high ammonium stress, the expression of *Low Phosphate Root 2* (*LPR2*), a gene encoding ferroxidase located in cell walls, was found to be up-regulated, which accelerated the transformation from Fe^2+^ to Fe^3+^, and ultimately led to the deposition of Fe^3+^ in the phloem. Fe^3+^accumulation resulted in an ROS burst, and was accompanied by callose accumulation, which inhibited the function of phloem and ultimately inhibited root elongation [25]. Coleto et al. [52] found that the double mutant of two MYB transcription factors, *myb28*and *myb29*, was very sensitive to ammonium stress. Under ammonium stress, the Fe content in stems was significantly lower than that in roots, while Fe in roots was higher than that in the wildtype, suggesting a defect of root-to-shoot Fe translocation in the mutant [52]. The above studies imply that high ammonium stress can disrupt Fe homeostasis in plants. However, this disruption is not directly caused by high NH_4_^+^, but rather by the impact of high ammonium stress on iron-related genes and enzymes, such as Fe transporters, *LPR2*, *MYB28*,and *MYB29*, as well as genes encoding proteins involved in Fe solubilization in the soil, like *BGLU42* (*β-Glucosidase42*), *F6H1* (*Feruloyl-Coa6-Hydroxylase 1*) and *FRO2* (*Ferric Reduction Oxidase 2*) [52]. On the other hand, reducing Fe supply may alleviate ammonium toxicity. Liu et al. [24] discovered that primary root growth retardation could be significantly alleviated when the Fe supply concentration was less than 50 μM. Li et al. [16] also reported that the accumulation of iron in plant tissues is a crucial factor leading to decreased ammonium nitrogen use efficiency (AUE) under high ammonium conditions. Appropriately reducing the iron content in Arabidopsis and lettuce under high ammonium conditions was beneficial for enhancing growth, nitrogen content, and nitrogen use index (UI) during the vegetative growth stage. Inhibiting ammonium-induced *LPR2* gene expression can reduce both iron accumulation and ammonium effluence, thereby improving plant AUE and growth under high ammonium conditions [16]. Coleto et al. [52] found that when *myb28* and *myb29* plants were grown with a higher Fe supply (200 μM vs 100 μM), the ammonium-sensitive phenotype was fully restored.

Besides Fe, high ammonium stress is also linked with other metal ions. Potassium (K^+^) is recognized as a vital macronutrient crucial for growth and development of plants [53]. Moreover, an adequate supply of K^+^ has been shown to improve the resilience of crop plants against a range of biotic and abiotic stresses, such as salt and drought [54]. Previous research has demonstrated that NH_4_^+^ reduces the primary influx of K^+^ from the external environment and suppresses its accumulation in plant tissues [55]. However, the detailed molecular mechanism by which high ammonium disturbs the homeostasis of K^+^ is not yet fully understood. Recent studies revealed that high NH_4_^+^ inhibited K^+^ absorption and induced NO (nitric oxide) accumulation through regulating *SNO1* (*Sensitive to Nitric Oxide 1*)/*SOS4* (*Salt Overly Sensitive 4*), thereby inhibiting primary root growth [23]. These findings suggest that high ammonium does not directly disturb K^+^ homeostasis, but rather affects the root development and inhibits K^+^ absorption consequently. On the other hand, an increase in K^+^ concentration can effectively alleviate ammonium toxicity [55,56,57,58,59,60,61]. Nevertheless, plant growth was impeded when the concentration of potassium reached 40mM, irrespective of the nitrogen source [55]. Balkos et al. [62] also showed that, in rice, increased K^+^ supply reduced futile NH_4_^+^ cycling at the plasma membrane, diminishing the excessive rates of both unidirectional influx and efflux. Similarly, this study also found that when the K^+^ concentration was too high (>40 mM), plant growth was hindered regardless of the N source [62]. The findings suggest that the maximal biomass is attained when NH_4_^+^ is used instead of NO_3_^−^, particularly in association with a moderately high supply of K^+^, indicating a preference of rice forNH_4_^+^ as a N source when K^+^ levels are optimized. Furthermore, decreases in root positive charges (Na^+^, K^+^, NH_4_^+^, Ca^2+^, Mg^2+^) and negative charges (Cl^−^, SO_4_^2−^ and PO_4_^3−^) were observed at high NH_4_^+^ availability [63] (Table 2).

### 3.4. High NH_4_^+^ Induced Disruption of Phytohormone Homeostasis

Recent studies have demonstrated that high ammonium can lead to disruptions in phytohormone levels [1,49]. The inhibition of root growth by NH_4_^+^ is associated with a reduction in free IAA content in the roots of Arabidopsis [32]. Di et al. [64] observed significant differences in NH_4_^+^ tolerance between two rice cultivars, Kas (Kasalath) and Kos (Koshihikari), attributing this disparity to the high capacity for auxin biosynthesis and superior maintenance of auxin levels under high ammonium stress in the NH_4_^+^-tolerant cultivar, Kos. Additionally, Di et al. [25] demonstrated that high NH_4_^+^ induced the expression of *WRKY46* in Arabidopsis, which subsequently led to the down-regulation of the primary root growth gene *NUDX9*(GDP-D-Mannose Pyrophosphohydrolase 9) and IAA-conjugating genes. This resulted in increased protein n-glycosylation in the root, leading to higher free IAA content and lower NH_4_^+^ efflux. However, when NH_4_^+^ stress was alleviated or removed, the accumulation of free IAA negatively regulated *WRKY46* expression and positively regulated IAA-binding gene expression, thereby maintaining low levels of IAA content in the roots [25]. Furthermore, the high NH_4_^+^ disturbs the subcellular IAA homeostasis by upregulating the expression of *PIN5* (PIN-formed 5). Knockout of *PIN5* resulted in elevated cytoplastic IAA accumulation and reduced NH_4_^+^ efflux under highNH_4_^+^ stress [26]. Furthermore, the coordinated signaling activity of auxin and brassinosteroids (BRs) is critical for optimal plant growth and development. NH_4_^+^ as the sole N source repressed BR signaling and response, which in turn inhibited auxin response and transport [65].

Ethylene has a negative effect on NH_4_^+^ tolerance in Arabidopsis. In the wild type, NH_4_^+^ stress enhanced the expression of *ACS* (*1-aminocyclopropane-1-carboxylic acid synthase*) and *ACO* (*1-aminocyclopropane-1-carboxylic acid oxidase*), the two key genes responsible for ethylene synthesis. After ethylene was perceived, the signal was transduced through the transcription factor EIN3. EIN3 regulated ROS accumulation, which led to oxidative stress in shoots under ammonium stress [29].

The involvement of ABA in ammonium toxicity has been noted. The plastid metalloprotease AMOS1/EGY1 (Ammonium Overly Sensitive1/Ethylene-dependent, Gravitropism-deficient, and Yellow-green-like protein1) were identified as key components in incorporating ABA into the ammonium signaling pathway in Arabidopsis. Transcriptome analysis revealed that 90% of the genes activated by ammonium were regulated by AMOS1/EGY1 [30]. Furthermore, a majority of AMOS1/EGY1-dependent and ammonium-activated genes contain a core motif of ABA-responsive elements in their promoters. Therefore, it was proposed that ammonium triggers a chloroplast retrograde signal leading to leaf chlorosis, while the AMOS1/EGY1-dependent response engages the ABA signaling pathway to protect leaves from chloroplast damage [30]. These results indicate that, although phytohormones are involved in ammonium toxicity, the disruption of phytohormone homeostasis may be a result of oxidative stress, rather than a direct reason for ammonium stress.

### 3.5. Rhizosphere Acidification

The process of the uptake of NH_4_^+^ leads to the enhancement of the activity of AMT-coupled PM H^+^-ATPases to exude H^+^ from the root cells to maintain a balanced cellular charge and prevent cytoplast acidification [7,66,67]. Therefore, the transport of NH_4_^+^ induces acidification of the rhizosphere and transient alkalinization of the cytosol [22,36,49]. Accordingly, studies have demonstrated that elevating the pH of the medium through the utilization of buffer solutions (Morpholineethanesulfonic acid, MES) or employing H^+^-ATPase inhibitor *N*,*N*’-dicyclohexylcarbodiimide can effectively mitigate the symptoms of ammonium toxicity [22,36].

**Figure 1:**
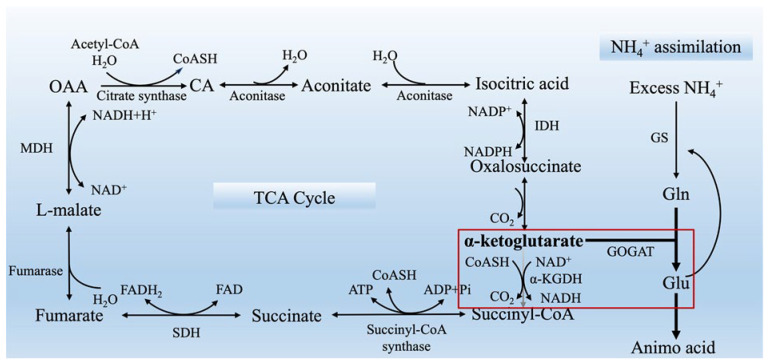
A schematic representation illustrating the assimilation of excessive NH_4_^+^ that results in the substantial consumption of intermediates from the TCA cycle, thereby causing an imbalance in C-N metabolism. α-KGDH,α-ketoglutarate dehydrogenase complex; ADP, adenosine diphosphate; CA, citrate; CoASH, coenzyme A; FAD, flavin adenine dinucleotide; Gln, glutamine; Glu, glutamate; GOGAT, glutamate synthase; GS, glutamine synthetase; IDH, isocitrate dehydrogenase; MDH, L-malate dehydrogenase; NAD, nicotinamide adenine dinucleotide; NADP, nicotinamide adenine dinucleotide phosphate; OAA, oxalacetic acid; SDH, succinate dehydrogenase. The red box illustrates that, under ammonium stress, an increased level of α-ketoglutarate is involved in the biosynthesis of Glu, leading to an imbalance in C-N metabolism.

NH_4_^+^ is primarily assimilated into amino acids through the glutamine synthetase (GS) and glutamate synthase (GOGAT) pathways. The activity of GS can be stimulated by the increased K^+^, which enhances the assimilation in the root of cucumber [45] and rice [60] and thus alleviates ammonium toxicity. Researches demonstrated that α-ketoglutarate and 2-oxaloacetate derived from the tricarboxylic acid (TCA) cycle play a crucial role in facilitating amino acid biosynthesis by providing components for the GS/GOGAT cycle to enhance NH_4_^+^ assimilation [10,68] (Figure 1). Additionally, studies have shown that supplementing with α-ketoglutarate can mitigate toxicity symptoms in tomato [69]. These findings suggest that enhancement of NH_4_^+^ assimilation may serve as a detoxification mechanism in plants. Hachiya et al. [21] also found that the excess assimilation process of ammonium in cells is the main cause of ammonium toxicity, which produces large quantities of H^+^, leading to acidification stress and plant growth inhibition. Moreover, the use of alkaline NH_3_ solution in the medium can reduce the acidification of cells and effectively alleviate the ammonium toxicity [21]. Similarly, Poucet et al. [70] also noted that the production of one Gln molecule through NH_4_^+^ assimilation mediated by GS results in the generation of two H^+^, leading to acidification. Furthermore, the symptoms of ammonium toxicity could be alleviated after the exogenous addition of L-methionine sulfoximine, an inhibitor of GS. Ma et al. [19] found that exogenous γ-aminobutyric acid (GABA) treatment limited NH_4_^+^ accumulation in rice seedlings, reduced NH_4_^+^ toxicity symptoms and promoted plant growth via inhibiting increases in GS/NADH-GOGAT activity when the concentration of NH_4_^+^ was more than 3 mM. GABA addition also reduced rhizosphere acidification and alleviated the inhibition of Ca, Mg, Fe and Zn absorption caused by excessive NH_4_^+^ [19] (Table 2).

However, a recent study has shown that rhizosphere acidification may not be the main reason for ammonium toxicity. Weil et al. [71] found that application of calcium carbonate buffering improved growth in solutions containing ammonium, but the plants did not restore their growth and nutrient accumulation to the levels achieved with solely nitrate nutrition. Similar results have been reported by many other studies [21,22,34,36,72]. These findings suggest that the acidification of the rhizosphere may not be the sole factor contributing to ammonium toxicity.

### 3.6. High NH_4_^+^ Induces Imbalance in C-N Metabolism

In plant cells, the TCA cycle initiates with the combination of acetyl-CoA and oxaloacetate to form citrate. The oxidation of citrate then produces reducing agents that facilitate adenosine triphosphate (ATP) synthesis through oxidative phosphorylation. Excessive amounts of NH_4_^+^ promote the biosynthesis of free amino acids, a process in which a large amount of C skeletons derived from the TCA cycle are consumed, thus leading to a C-N imbalance (Figure 1), as observed in rice [49,73]. Du et al. [42] reported that the solely ammonium treatment significantly decreased root growth, protein content and the concentrations of most intermediates and the activity of enzymes from the TCA cycle. Additionally, the ammonium treatment increased the activities of invertase, sucrose synthase, and trehalose 6-phosphate synthase, which accelerated glycolysis. Then, substantial quantities of carbohydrates were remobilized from leaves to roots to sustain the energy expenditure needed for NH_4_^+^ assimilation [73]. An early study showed that, in the ammonium-sensitive crop barley, high levels of external ammonium induced a significant NH_4_^+^ efflux, and such futile NH_4_^+^ cycling placed an energy burden (carbohydrate consuming) on plant growth [74]. These results indicate that the imbalance in C-N metabolism caused by ammonium stress may be one of the main reasons for ammonium toxicity. Furthermore, the relationships between ion imbalance, disruption of phytohormone homeostasis, accumulation of ROS, and C-N metabolism imbalance warrant further investigations (Table 2).

**Table 2:** Effects of external and intrinsic regulators on plant response to high-NH_4_^+^ stress.

Regulator	Pathways/Mechanisms	Species	Reference
NO_3_^−^	Nitrate interacts with NRT1.1 to promote NO_3_^−^ cycling across the membrane.	Arabidopsis	[22]
	Nitrate inhibits acidification and promotes NH_4_^+^ assimilation.	oilseed rape	[43]
	Nitrate modulates phytohormone pathways.	wheat	[40]
ROS	ROS scavengers reduce ROS deposition in the phloem.	Arabidopsis	[24]
	VB6 reduces H_2_O_2_ accumulation upon ammonium toxicity.	Arabidopsis	[47]
	Heme oxygenase OsSE5boosts the activities of ROS-scavenging enzymes.	rice	[48]
	Ammonium toxicity inhibits photosystems and electron transfer, thus inducing ROS accumulation.	oilseed rape	[35]
Iron	The cell wall-localized ferroxidase LPR2leads to Fe and callose deposition in the phloem.	Arabidopsis	[24]
	High NH_4_^+^-induced iron accumulation triggers excess NH_4_^+^ efflux.	Arabidopsis, lettuce	[16]
	The *myb28,myb29* double mutant shows altered Fe accumulation and is highly hypersensitive to ammonium nutrition.	Arabidopsis	[52]
Potassium	K^+^ competitively inhibits the uptake and accumulation of NH_4_^+^ and optimizes NH_4_^+^ metabolism.	rice	[55]
	K^+^ leads to higher carbon and energy availability and improves ion homeostasis.	pea	[63]
	K^+^ supply reduces futile NH_4_^+^ cycling at the plasma membrane.	rice	[62]
	Increase in K^+^ concentration can effectively alleviate ammonium toxicity.	Arabidopsis, rice, barley	[55,56,57,58,59,60,61]
Phytohormone	Ammonium toxicity decreases free IAA content in roots by inhibiting the transcription of auxin-biosynthesis genes.	rice	[64]
	WRKY46promotes ammonium tolerance by repressing IAA-conjugating genes.	Arabidopsis	[25]
	High NH_4_^+^ disturbs the subcellular IAA homeostasis by upregulating the expression of PIN5.	Arabidopsis	[26]
	Ammonium toxicity repressing BR signaling, thus inhibiting auxin response and transport.	Arabidopsis	[65]
	Plants over-expressing EIN3 (a key regulator of ethylene responses) are more sensitive to NH_4_^+^toxicity.	Arabidopsis	[29]
	ABA signaling is required for the regulation of expression of NH_4_^+^-responsive genes.	Arabidopsis	[30]
Rhizosphere pH and the TCA cycle	AMTs enhance the activity of AMT-coupled H+-ATPases to exude H^+^ from the root cells.	Arabidopsis, rice	[7,66,67]
	Medium buffer MES and *N*,*N*′-dicyclohexylcarbodiimide elevate medium pH and inhibit H^+^-ATPase activity.	Arabidopsis	[22,36]
	α-ketoglutarate and 2-oxaloacetate furnish components for the GS/GOGAT cycle to promote NH_4_^+^ assimilation, thus preventing NH_4_^+^ toxicity.	Arabidopsis, *Lycopersicon esculentum*, *Myriophyllum aquaticum*	[10,68,69]
	Application of an alkaline solution efficiently alleviates ammonium toxicity with a concomitant reduction in shoot acidity.	Arabidopsis	[21]
	GABA limits NH_4_^+^ accumulation, inhibits increases in GS/NADH-GOGAT activity and reduces rhizosphere acidification caused by excessive NH_4_^+^.	rice	[19]
	Through synergistic activation of the NRT1.1-SLAH3 complex, efficient transmembrane cycling of NO_3_^−^ is induced, effectively inhibiting rhizosphere acidification.	Arabidopsis	[36]
C-N metabolism	Upon ammonium toxicity, increased activities of invertase, sucrose synthase, and trehalose 6-phosphate synthase leads to enhanced glycolysis and a significant energy expenditure.	rice	[73]
	Insufficient sucrose distribution caused by impaired phloem function under high ammonium stress is the reason for the inhibition of root elongation.	Arabidopsis	[24]
	SnRK1.1 works upstream of SLAH3 to regulate hypocotyl growth during skotomorphogenesis in response to changes in sugar levels induced by ammonium toxicity.	Arabidopsis	[22]

**Figure 2:**
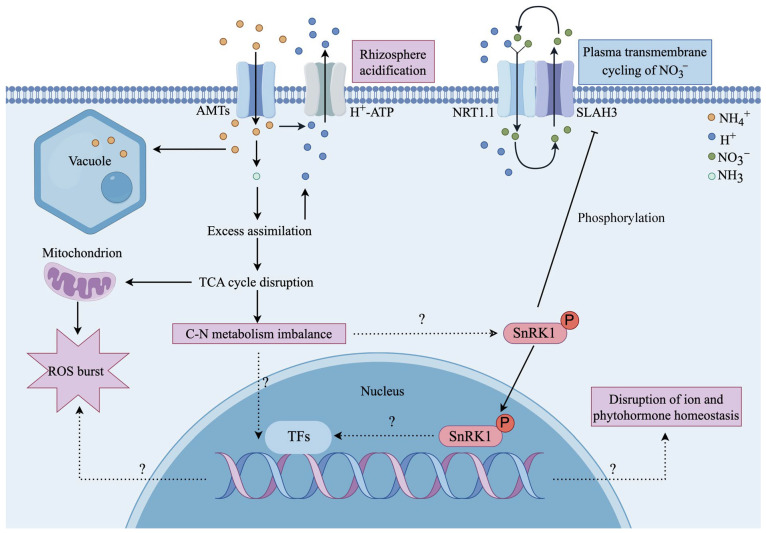
A proposed model elucidating the role of SnRK1 in ammonium toxicity. The excessive uptake and assimilation of NH_4_^+^ result in an increased production of H^+^ via H^+^-ATPase, which subsequently leads to the acidification of the rhizosphere and inflicts cellular damage and toxicity. Furthermore, the over-assimilation of NH_4_^+^ depletes numerous intermediates from the TCA cycle, thereby disrupting the balance of C-N metabolism. However, the relationships between metabolic imbalance and ion imbalance, disruption of phytohormone homeostasis and accumulation of ROS remain inadequately understood. Under normal conditions, SnRK1.1 phosphorylates SLAH3 to inhibit its activity; under high-ammonium conditions, SnRK1.1 migrates to the nucleus, which releases the inhibition on SLAH3 and leads to nitrate efflux. A potential role of the energy sensor SnRK1 in C-N metabolism imbalance under ammonium stress has been proposed; however, the downstream transcription factors (TFs) still need to be identified. AMT, ammonium transporters; NRT1.1, nitrate transporter 1.1; ROS, reactive oxygen species; SLAH3, Slow anion channel-associated 1 homologue 3; SnRK1, SNF1-related protein kinase 1; TCA, tricarboxylic acid.

The SNF1-related protein kinase 1 SnRK1, a crucial regulator of C and energy metabolism in plants, is analogous to the yeast SNF1 (Sucrose Non-Fermenting-1) and mammalian AMPK (Adenosine-Monophosphate-activated Protein Kinase). This eukaryotic AMPK/SNF1/SnRK1 protein kinase is commonly composed of a heterotrimeric complex comprising an α catalytic subunit (including KIN10, KIN11 and KIN12 in Arabidopsis) and two regulatory subunits (β and γ) [37,75,76]. Besides a large number of reports in yeast and mammals, many researchers have also studied the role of SnRK1 in plants. As an important energy sensor in plants, SnRK1 participates in many physiological processes of plants, such as flowering [37,75,76] and stress responses [77]. However, the participation of SnRK1 in addressing ammonium toxicity has not been extensively documented. Sun et al. [22] demonstrated that under high ammonium stress, SnRK1.1 (KIN10) translocated from the cytoplasm to the nucleus, where it accumulated and relieved the inhibition of SLAH3. The resulting NO_3_^−^ efflux through SLAH3 helps alleviate rhizosphere acidification stress induced by high NH_4_^+^ (Figure 2). Interestingly, sucrose also plays a role in ammonium toxicity, with 0.5% sucrose leading to the most significant recovery of an ammonium-stressed phenotype [22]. These researchers found that the *SnRK1.1* over-expression lines did not exhibit phenotype differences compared to the wild type when grown without sucrose; the over-expression lines showed significantly shortened hypocotyls with 1% sucrose in Col-0 background, but not in *slah3-4* background, indicating that SnRK1.1 works upstream of SLAH3 to regulate hypocotyl growth during skotomorphogenesis in response to sugar signals [22]. Liu et al. [24] also reported the relationship between sucrose and ammonium toxicity. They found that the insufficient sucrose supply caused by impaired phloem function under high ammonium stress inhibited root elongation. Insufficient sucrose supply not only directly leads to C depletion but also results in failure to meet the energy demands for futile transmembrane NH_4_^+^ cycling, also indicating the C-N metabolism imbalance in ammonium toxicity [78,79]. Furthermore, SnRK1 has been suggested to be a major energy sensor in plants and has been reported to be activated by carbon deficiency stresses [80]. During carbon deficiency or nitrate depletion, SnRK1.1 phosphorylates the transcription factor NLP7 (NIN-like protein 7) at Ser-125 and Ser-306 and promotes its degradation, therefore repressing nitrate-responsive gene expression and inhibiting plant growth [81] (Figure 2). However, the *nlp7* mutant showed a similar sensitivity to ammonium toxicity, compared with the wild-type plants [82]. Thus, downstream of SnRK1.1, the key transcription factors responsive to ammonium toxicity still need to be identified.

### 3.7. High NH_4_^+^ May Change Rhizosphere Microorganisms

The benefits of plant growth-promotingmicroorganisms (PGPMs) as plant inoculants areinfluenced by a wide range of environmental factors, including ammonium [83]. The microbial consortia product (MCP) inoculants stimulated root and shoot growth and improved the acquisition of macronutrients only on a freshly collected field soil with high organic matter content, exclusively in combination with stabilized ammonium fertilization [83]. When the nitrate fertilization was replaced by ammonium, five out of seven investigated phosphate-solubilizing microorganisms (PSM) inoculants exerted beneficial effects on plant growth and reached up to 88% of the shoot biomass production of a control supplied with soluble triple-superphosphate [84]. Whether excessive ammonium may be toxic to some rhizosphere bacteria or reduce the metabolites from these bacteria that arenecessary for plants needs further investigations. So far, we cannot conclude that alternations in PGPMsis one of the reasons for ammonium toxicity. Nevertheless, this is an interesting future direction for research.

## 4. Perspectives on Future Studies

Ammonium is the preferred N source for most plants. Yet when utilized as the exclusive nitrogen source, it can impede growth. Despite numerous studies investigating the mechanisms of ammonium toxicity, the primary reasons for plant growth inhibition under high ammonium stress are still not fully understood. Excessive use of fertilizers has led to considerable environmental contamination and has negative consequences for the growth and yield of crops, vegetables, and fruits. Consequently, it is crucial to comprehend the precise mechanisms of ammonium toxicity and then devise efficient mitigation strategies to enhance agricultural productivity.

Signs of ammonium toxicity do not appear as a singular symptom but as complex phenotypes on the whole seedling. Nevertheless, numerous researchers focus exclusively on root elongation, disregarding alterations in stems, leaves, and total biomass. In this review, we suggest a comprehensive assessment to determine the severity of ammonium toxicity.

Some plants (e.g., Arabidopsis) show lethal phenotypes when grown under solely high-level ammonium conditions. Therefore, a large number of studies adopted treatments with high ratios of ammonium to nitrate. Nevertheless, the toxic mechanisms of a high ratio of ammonium to nitrate may be much different from those of a solely ammonium treatment. Here we suggest that genes/proteins identified from high-ratio conditions should be verified under the solely ammonium condition.

Ammonium stress commonly results in the accumulation of ROS [24,35,46,47,48,49], just as other environmental stresses do, and numerous studies have demonstrated that high ammonium causes disturbances in ion levels [16,52,53,54,55,56,57,58,59,60,61,62,63] and phytohormones homeostasis [25,26,27,28,29,30,64,65]. However, the disruption of phytohormone homeostasis may be a result of oxidative stress, rather than a direct reason for ammonium stress. A recent review has suggested that ROS generation is a downstream response to C-N metabolism disruption induced by ammonium stress, indicating that the accumulation of ROS is not the primary cause of ammonium toxicity [49].

The uptake of ammonium by root cells and its subsequent assimilation lead to the generation of a significant quantity of H^+^, causing acidification of the rhizosphere and potential damage to cell walls and membranes. However, enhancing pH value by exogenous chemical treatments did not result in a complete recovery of plant growth under high ammonium conditions [21,22,34,36,71,72]. This implies that rhizosphere acidification may be only a contributing factor to ammonium toxicity. Early studies showed that the transmembrane transport of NH_4_^+^ consumes a lot of energy, while recent studies concluded that the process of NH_4_^+^ entering cells under high ammonium is a passive transport pathway, which does not consume energy, and NH_4_^+^ can be directly incorporated into C chains without a process of reduction [49,85]. Thus, excessive energy expenditure may also not be the main reason for ammonium toxicity.

Moreover, high ammonium was found to disrupt the TCA cycle and cause a C-N metabolic imbalance [42,86], which is thought to be one of the primary causes of ammonium toxicity [49]. However, it should be noted that, besides high NH_4_^+^, high NO_3_^−^also induces an imbalance in C-N metabolism, as nitrate is ultimately reduced to ammonium through nitrate reductase and nitrite reductase for its assimilation [37,76]. The ammonium toxicity cannot be only attributed to C-N metabolism imbalance. An application of extra C to regulate the C:N ratio cannot mitigate ammonium toxicity completely [86].

SnRK1 has been suggested to be involved in this regulatory pathway. Nevertheless, despite its significance as a crucial regulatory factor in plants under environmental stresses, there is limited literature indicating the role of SnRK1 in ammonium toxicity and the direct experimental evidence is still lacking. SnRK1 mainly includes two subunits, KIN10 and KIN11. They exhibit functional redundancy, and the transgenic Arabidopsis over-expressing *KIN10* only showed a week phenotype under the ammonium stress [22]. On the other hand, the *kin10*, *kin11* double mutant is lethal [87]. Therefore, conditional (chemical-inducible) knockdown mutants [88] may be constructed or VIGS (Virus Induced Gene Silencing) technology [87] may be adopted.

In future research, the identification of genes associated with ammonium toxicity, methods of comparative transcriptomics may be a focus. For example, a pair of plants preferring NH_4_^+^ and NO_3_^−^, respectively, may be selected. Both plants could then be subjected to a high ratio of ammonium to nitrate or a solely ammonium treatment. Then genes expressed more inNH_4_^+^-preferring plants than in NO_3_^−^-preferring plants could be identified by RNA-seq, and these genes may be associated with the tolerance to ammonium toxicity. Nevertheless, as mentioned above, genes/proteins identified from the high-ratio condition should be verified under the solely ammonium condition.

## 5. Conclusions

Most previous studies paid attention to root morphological change under high ammonium treatments, but not growth inhibition or development arrest of the whole plant seedling. In this review, we summarized multiple adverse effects of excessive ammonium on the whole plant seedling and suggest that both root phenotypes and shoot phenotypes should be considered when determining the severity of ammonium toxicity.

Previous studies demonstrated that ROS burst, rhizosphere acidification, disruption of metal ion homeostasis and phytohormone homeostasis are the key mechanisms of ammonium toxicity. However, through our analysis, we conclude that none of them are the direct reasons for ammonium toxicity. Furthermore, we point out that the mechanisms of toxicity caused by a high ratio of ammonium to nitrate and the solely ammonium treatment may be significantly different. C-N metabolism imbalance plays a more important role in high ammonium stress response, where the signaling role of SnRK1warrants further investigations.

## Figures and Tables

**Table 1:** A comprehensive assessment to the impacts of ammonium toxicity.

Tissues	Symptoms	NH_4_^+^ Concentration	Species	References
Roots	Inhibition of primary root elongation	5–30 mM	Arabidopsis, rice	[24,25,26,31]
	Modifications in lateral roots, reduced root/shoot ratio and diminished fresh root weight	5–80 mM	Arabidopsis	[21,22,27,28]
Stems	Inhibition of stem growth and reduced stem fresh weight	10–50 mM	Arabidopsis	[21,29]
Leaves	Leaf chlorosis	20–60 mM	Arabidopsis, barley	[30,31,32]
Flowers and seeds	Reduced plant biomass and yield	5–20 mM	Arabidopsis, oilseed rape	[21,22,33,34,35,36]
	High NH_4_^+^-induced late flowering	40 mM	Arabidopsis	[37]
Whole plant	Whole seedling death	>1 mM	*Arnica montana*, *Cirsium dissectum*, *Calluna vulgaris*	[4,10,38]
